# A design principle for tuning far-red absorption of chlorophyll *a* in light-harvesting complexes

**DOI:** 10.1038/s42004-026-02052-0

**Published:** 2026-05-04

**Authors:** Alessandro Agostini, Eduard Elias, Niccolò Cianfarani, Tim J. Dalebout, Donatella Carbonera, Roberta Croce

**Affiliations:** 1 Department of Chemical Sciences, University of Padova, Padova, Italy; 2 Biophysics of Photosynthesis, Department of Physics and Astronomy, Faculty of Science, Vrije Universiteit Amsterdam and LaserLaB Amsterdam, Amsterdam, the Netherlands

**Keywords:** Enzyme mechanisms, Biophysical chemistry, Proteins, Photobiology

## Abstract

Far-red absorption in eukaryotic light-harvesting complexes (LHCs) has been associated with strongly excitonically coupled chlorophyll *a* clusters exhibiting mixing with charge-transfer states, yet the structural rules enabling this spectral tuning remain unclear. Previous studies have highlighted the importance of the amino acid ligating Chl *a*603 in providing the pigment orientation required for the formation of a red-shifted Chl *a*603-*a*609 cluster. More recently, it has been suggested that the steric properties of the residue at the *i*-4 position from the ligand may also play a crucial role.

Here, we test this hypothesis through targeted mutagenesis of two light-harvesting complexes, Lhca4 and CP29, which host the Chl *a*603-*a*609 pair, but differ in their protein environment and spectral properties. In Lhca4, introduction of steric constraints at the *i*-4 position relative to the Chl *a*603 ligand (A43L) abolishes far-red absorption, indicating that steric crowding at this position destabilizes the strongly coupled pigment configuration. In CP29, substitution of the Chl *a*603 ligand (H111N) is required for far-red absorption, while the additional mutation at *i*-4 position (H111N/C107A) modulates the magnitude of the red-shift. Together these results highlight the importance of both axial ligand identity and local protein environment in controlling far-red absorption in Chl *a* clusters.

## Abstract (web summary)

Far-red absorption in eukaryotic light-harvesting complexes is attributed to excitonically coupled chlorophyll a clusters, yet the structural basis for this spectral tuning remains unclear. Here, the authors use targeted mutagenesis on two light-harvesting complexes Lhca4 and CP29 which host the Chl a603-a609 cluster, revealing that both axial ligand identity and local protein environment play key roles in modulating far-red spectral tuning.

## Introduction

The biosphere relies mainly on sunlight for its energy, which photosynthetic organisms capture via light-harvesting complexes. These are proteins that bind pigments, allowing effective light-harvesting while providing photoprotective channels for dissipating excessive energy^1,2^. Photosynthetic eukaryotes rely mainly on chlorophylls and carotenoids to capture light energy but they extend their spectral coverage through fine-tuning of chromophore-chromophore and chromophore-protein interactions. Numerous studies have addressed the contributions of individual amino acids to modulate the properties of the bound (bacterio)chlorophylls by (i) determining their three-dimensional arrangement^3–8^, (ii) providing hydrogen-bonds^4,9–18^, (iii) altering the steric hindrance^19–22^ and/or the electrostatic potential^18,21,23–29^ of their surroundings.

**Fig. 1:**
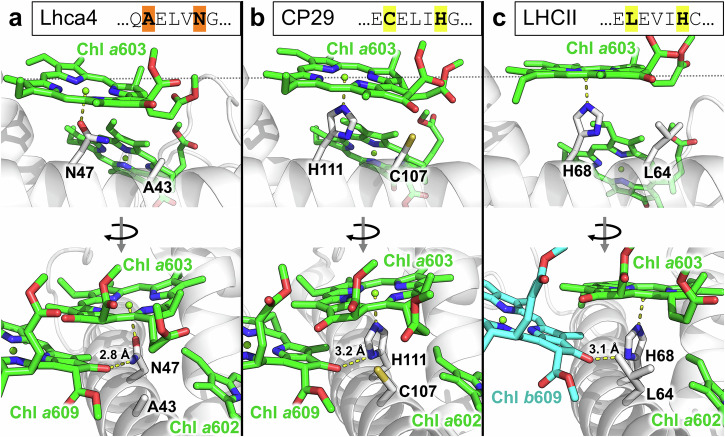
Comparison of the Chl *a*603 binding site in relevant LHCs. Detailed view of the chlorophyll-binding site *a*603 in (**a**) *Pisum sativum* Lhca4 (PDB ID: 7DKZ^58^), and *Spinacia oleracea* (**b**) CP29 (PDB ID: 3PL9^59^) and (**c**) LHCII (PDB ID: 1RWT^36^). Green sticks, Chls *a*; cyan sticks, Chl *b*; white cartoons, polypeptide chain, white sticks, amino acids.

A particularly interesting system is the one constituted by Photosystem I (PSI)-specific antenna complexes Lhca3 and Lhca4, which display the red-most absorption and emission spectra among plant LHCs^30,31^. These complexes exhibit Chl *a* spectral forms having remarkably red-shifted absorption and fluorescence spectra^32–34^, with emissions up to 750 nm in some shade-adapted plants of the Acanthaceae family^35^. It was shown that Chl *a*603 and *a*609 (nomenclature from Liu et al.^36^) associated with Lhca3 and Lhca4 are responsible for this absorption^3,37^ (Fig. 1a). This red-shift is attributed to a mixing between the excited and charge-transfer states within this tightly packed Chl *a* dimer^7,38–41^, resulting in broadened absorption and emission bands with a large Stokes shift^40,42^. Recently, a role of the *a*602-*a*603 dimer has also been proposed^43^. It was previously shown that the identity of the amino acid ligation of Chl *a*603^3,37^ is crucial to correctly orient^7,8^ these two pigments to originate markedly red-shifted excitons characterized by a significant charge transfer character^39^. The presence of an asparagine as a ligand of Chl *a*603 has therefore been considered an important signature of red forms in Lhcs across eukariotic organisms^44–49^.

Recently, some of us have proposed that the presence of a small sidechain residue at the *i*-4 position from the ligand of Chl *a*603 is also necessary for far-red absorption^50^. This [A/G]xxxN motif was proposed not only to be the basis the red forms found in antennae linked to PSI, but also to impart the far-red absorption capability found in the antennae linked to PSII in microalgae such as diatoms^48,51,52^ and eustigmatophytes^49,53–55^ (a recent study from Seki et al^56^. assigned the red form of the latter to a large cluster of seven excitonically coupled Chls *a*, comprising a Chl *a*609 bound through a [A/G]xxxN ligation motif). Latterly, the [A/G]xxxN motif was also identified in the PSII antennae of the Antarctic green alga *Prasiola crispa*^57^, which are characterized by red forms^44^.

The present work aims to test the role of the *i*-4 residue in the far-red absorption in LHCs, by means of a site-directed mutagenesis approach. On the one hand, we introduced a bulky amino acid at position *i*-4 from the *a*603 binding site in Lhca4 to test its ability to alter the red form (A43L mutation, following the notation of Wang et al.^58^), on the other hand we selected CP29 as a model system in which introducing a red form by means of the simultaneous exchange of the amino acid ligating Chl *a*603 and of the amino acid at position *i*-4 from it (H111N and C107A mutations, respectively, following the notation of Pan et al.^59^). The choice of CP29 derives from its monomeric nature and the occupancy of site 609 with a Chl *a*, as in Lhca4 (contrarily to LHCII, see Fig. 1). A comprehensive biochemical and spectroscopic analysis was conducted to assess the impact of the mutations on their overall light harvesting characteristics.

## Results and discussion

### Sample preparation

As shown previously, both Lhca4 and CP29 can be reconstituted in vitro with a suitable pigment mixture to yield complexes with a pigmentation that is very similar to that of the corresponding native complexes^4,60^. In the present study, both WT and mutant proteins were expressed, purified, and reconstituted in vitro under identical conditions to ensure direct comparability. Chl *a*/*b* and Chl/Car ratios of the reconstituted samples are reported in Table 1.

**Table 1:** Pigment composition

Sample	Chl/Car	Chl *a*/*b*
Lhca4 WT	4.8	2.0
Lhca4 A43L	5.0	2.0
CP29 WT	3.6	1.9
CP29 H111N	3.4	1.9
CP29 C107A/H111N	3.3	1.9

Chl *a*/*b* and Chl/Car ratio of Lhca4 and CP29 samples, based on three repetitions. The standard deviation (SD) is < 0.2.

In Lhca4 WT, the Chl/Car ratio and the Chl *a*/*b* ratio are very similar to those previously reported^4,61^ The Chl/Car and Chl *a*/*b* ratios in the A43L variant closely match those of the WT, indicating that the introduction of the bulky amino acid does not disrupt the binding of chlorophylls in the nearby *a*603 and *a*609 binding sites, nor in any other site.

Similarly, in all CP29 variants, the Chl/Car and Chl *a*/*b* ratios are consistent with earlier reports^60,62^. The Chl/Car ratio remains largely unaffected by the mutation, indicating that none of the modifications result in chlorophyll loss.

### The A43L mutation leads to a disruption of the red form of Lhca4

**Fig. 2:**
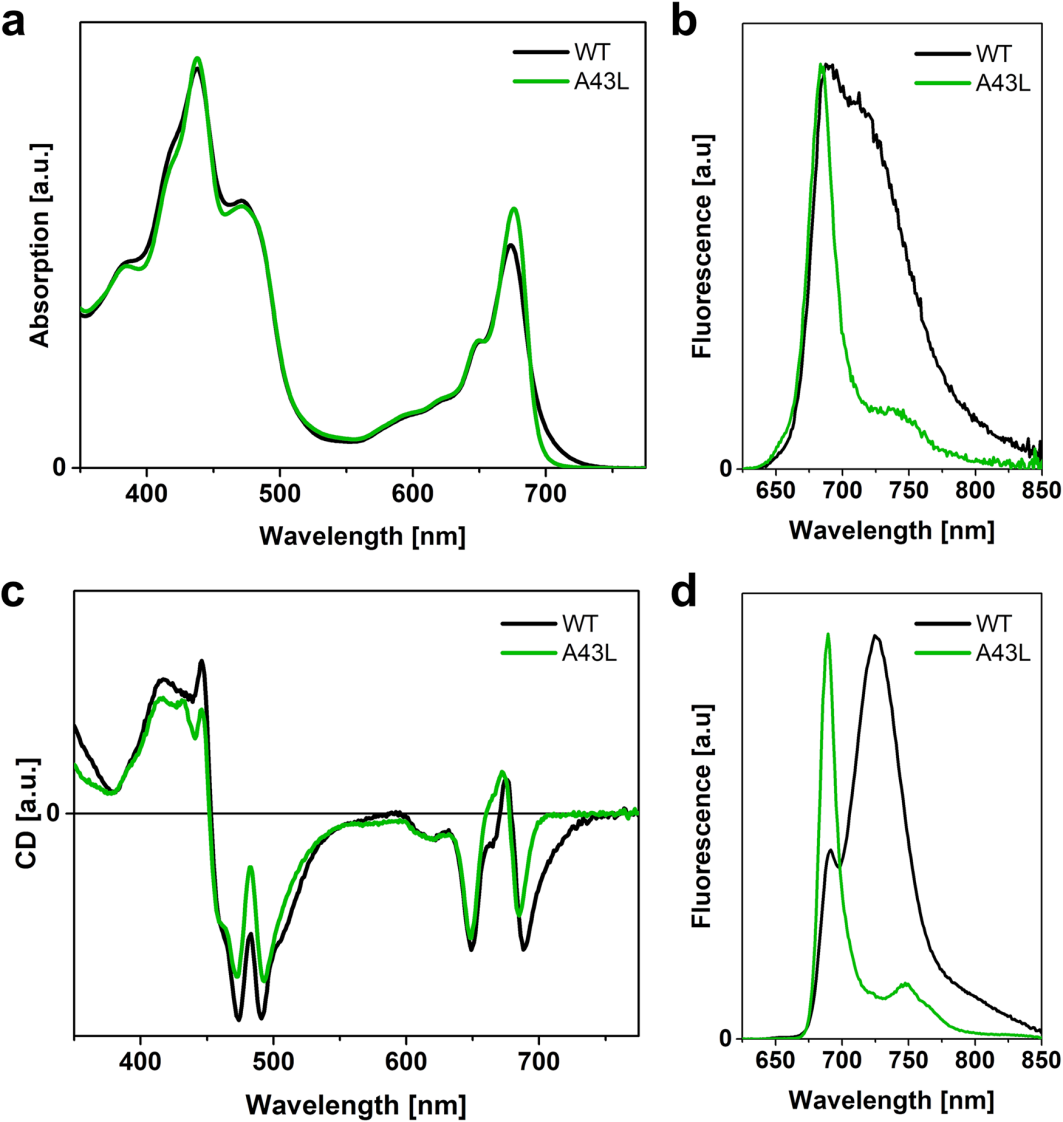
Steady-state spectra of Lhca4 WT and A43L mutant. Absorption (**a**), fluorescence (**b**, **d**), and CD (**c**) spectra of Lhca4 WT (black) and A43L (green). Emission spectra recorded after excitation at 500 nm. Spectra have been measured either at RT (**a**, **b**), 277 K (**c**), or 77 K (**d**). Absorption and CD spectra are normalized to the same integrated area in the Q-region (630-750 nm), the fluorescence spectra are normalized to their maxima.

Figure 2 shows the RT absorption spectra of the Lhca4 complexes. In the Q-region large differences can be appreciated: in the A43L mutant the absorption above 700 nm is suppressed, while it increases at its maximum (673 nm). This behavior is the same as previously observed in the case of the Lhca4 N47[H/Q] mutants^3,4,8^, and can similarly be attributed to the absorption of “monomeric” Chls *a*603 and *a*609 in the absence of the strong excitonic interaction/charge transfer character of their WT arrangement, in agreement with previous results. The small residual absorption tail beyond 700 nm is at the same level observed in other LHCs that do not contain red forms, such as LHCII (Fig. S2a). Static disorder may occasionally favor pigment configurations that still allow some degree of exciton–CT mixing in the *a*603-*a*609 dimer, so that some fraction of complexes would always exhibit some far-red absorption, consistent with earlier single-molecule studies on Lhca4^63^ and related LHCs^64–68^.

The role of the A34L mutation in disrupting the red form of Lhca4 is even clearer when the RT and 77 K emission spectra of the A43L mutant are analyzed (see Fig. 2b, d and S2b), where the far-red emission is completely lost, analogously to the N47H mutant^3,4^.

The loss of the red forms is confirmed by the CD spectrum (Fig. 2c), where marked differences are observed in the red part (λ > 700 nm) of the spectrum where the mutant clearly lacks the negative signal that arise from the excitonic interaction responsible for the red forms^3^, a result similar to what previously observed in the case of the N47[H/Q] mutants^4,8^. In the Soret region, the A43L CD spectrum is characterized by a very similar profile to that of the WT, indicating that the mutation does not change the overall pigment organization of the complex.

### The C107A mutation slightly red-shifts the absorption of CP29

**Fig. 3:**
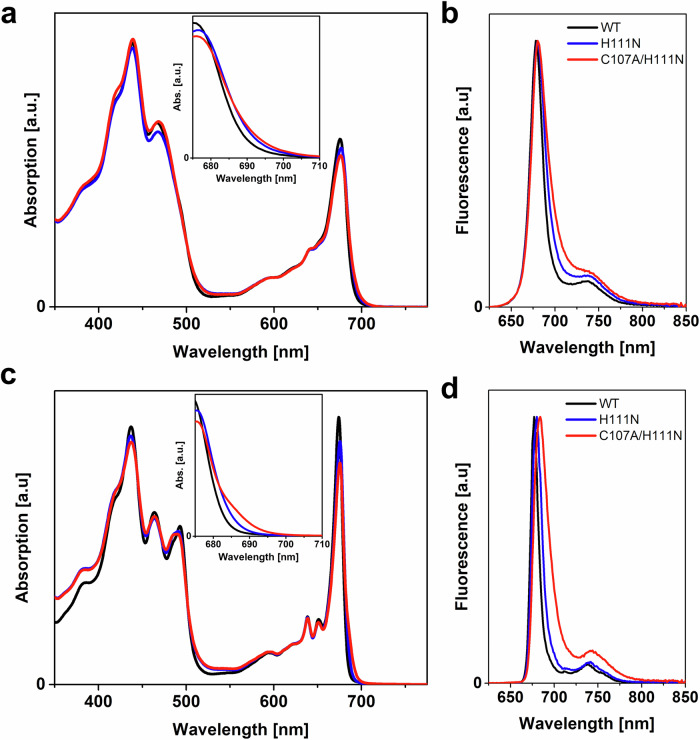
Steady-state spectra of CP29 WT, H111N and C107A/H111N mutants. Absorption (**a**, **c**) and fluorescence (**b**, **d**) spectra of CP29 WT (black), H111N (blue), and C107A/H111N (red). Emission spectra recorded after excitation at 500 nm. Spectra have been measured either at RT (**a**, **b**) or at 77 K (**d**). Absorption spectra are normalized to the same area in the Q region (600–750 nm), the fluorescence spectra are normalized to their maximum.

In the case of CP29, the analysis of the Q-region of their absorption spectra reveals a slight increase in the absorption above 700 nm in the mutants. The slight red-shift of the absorption observed in the H111N mutant^5,6^ is further enhanced by the additional C107A mutation, resulting in a red shoulder that becomes partially resolved in the 77 K spectrum of the CP29 C107A/H111N double mutant (see inset in Fig. 3c). The cumulative effect of the two mutations is even clearer when the emission spectra of the complexes are analyzed. The spectra got wider and shifted to the red in the CP29 WT-H111N-C107A/H111N series, suggesting a progressive stabilization of a red-shifted state.

In the CD spectra of the CP29 mutants a slight increase emerges in the far-red spectral region (λ > 700 nm), congruently with the corresponding shoulder in the absorption spectra (Fig. 3). Besides that, overall their CD spectra closely resemble that of the WT (Fig. S1), suggesting that these mutations have no major impact on the excitonic interactions or pigment arrangement.

**Fig. 4:**
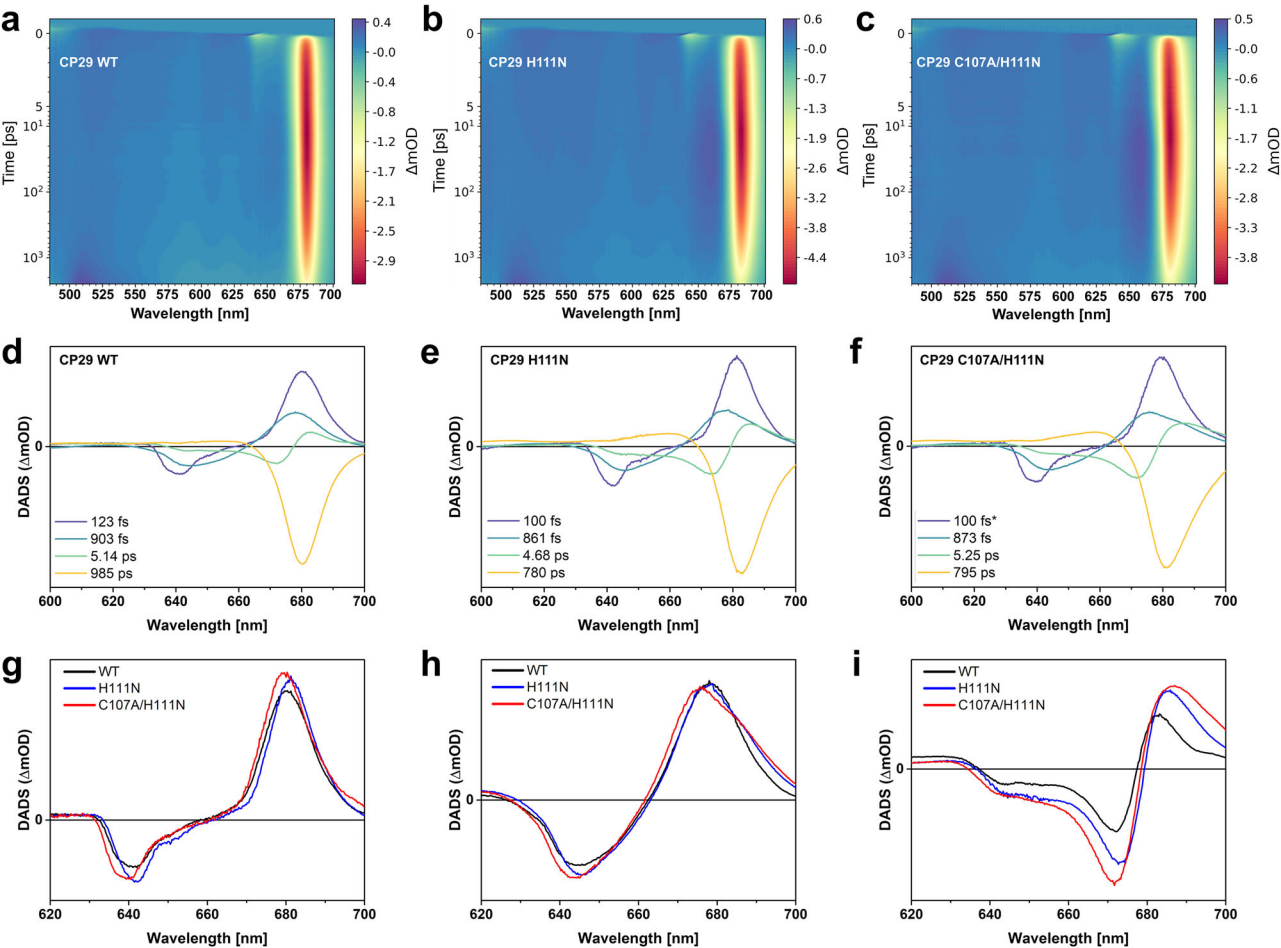
Transient absorption data for CP29 WT, H111N, and C107A/H111N excited at 642 nm. Spectrotemporal maps of CP29 WT (**a**) H111N (**b**) and C107A/H111N (**c**). DADS of CP29 WT (**d**), H111N (**e**), and C107A/H111N (**f**, for an extended range see Fig. S3). The star (*) marks a fixed lifetime. The (**g**) first, (**h**) second, and (**i**) third DADS components are compared to better highlight the red shift trend in the CP29 WT (black), H111N (blue), and C107A/H111N (red) series.

To investigate the impact of the mutations on energy transfer pathways and kinetics of CP29, we conducted ultrafast transient absorption and time-resolved fluorescence measurements at room temperature. In all transient absorption experiments, the excitation wavelength was set to 642 nm to preferentially excite Chls *b*. The resulting spectro-temporal maps are shown in Fig. 4a–c. A quantitative analysis of energy transfer dynamics was achieved through global fitting of the data. Four components were sufficient to satisfactorily describe the data (see Figs. S4–6).

The spectral evolution of the three samples is better evaluated from the analysis of the Decay-Associated Difference Spectra (DADS) (Fig. 4d–f) obtained through the global analysis of the maps (reported in Fig. 4a–c). The first and second components, dominated by the energy transfer from Chls *b* (640–650 nm) to Chls *a* (670–685 nm), have similar lifetime and spectra in all three CP29 variants, with peak red-shifts and a slight increase in the red tail that follow the trend WT < H111N < C107A/H111N (Fig. 4g, h). The third component, with a time constant of about 5 ps, shows a red-shifted and broader positive feature (Fig. 4i). This component can be assigned to energy transfer to lower-energy Chls *a* and follows a marked WT < H111N < C107A/H111N trend in both its relative intensity and its red-shift, consistent with the pattern observed in the steady state measurements (Fig. 3).

Taken together, the data point to an unambiguous red-shift of the low-energy Chl *a* forms, confirming that the C107A mutation concur, along with the H111N one, in altering the arrangement of the Chls *a* in a way that red-shift the redder excitonic state. Moreover, the relative magnitude of this component is significantly larger in both mutants than in the WT, showing that a larger fraction of excitation energy to the lower energy sites is transferred within this timeframe.

**Fig. 5:**
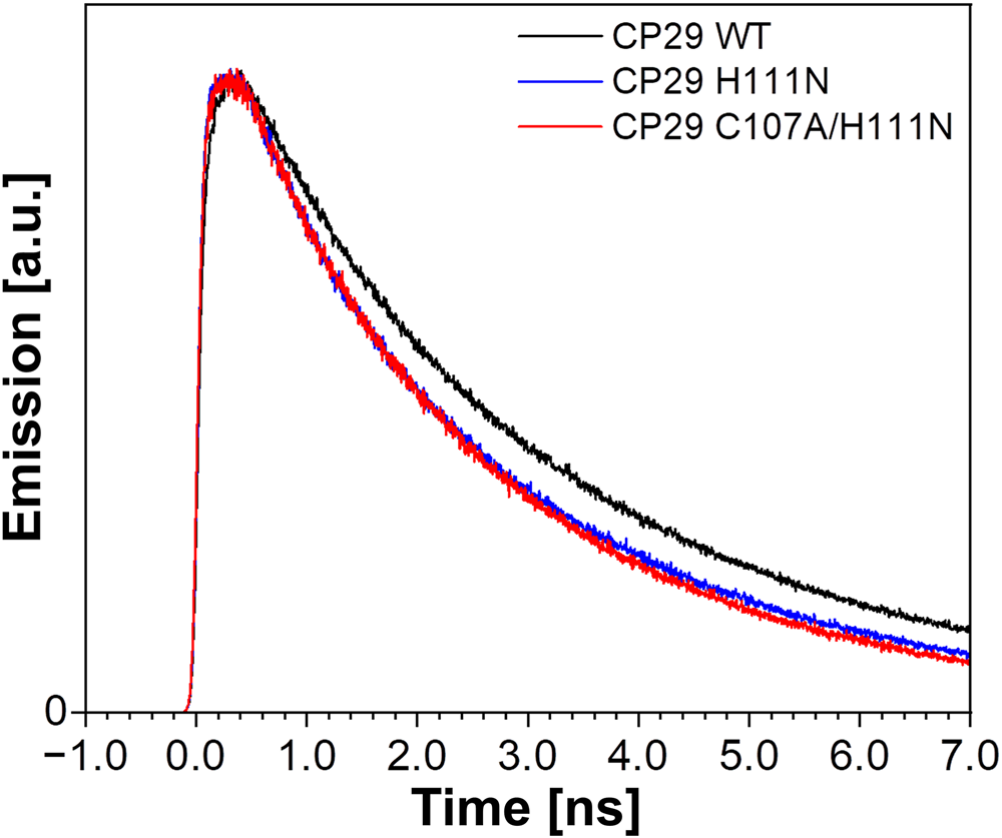
Time-resolved fluorescence. TCSPC data for CP29 WT (black), H111N (blue), and C107A/H111N (red) excited at 466 nm and detected at 680 nm.

The excited state decay of the three samples has been evaluated by measuring their time-resolved fluorescence using a Time Correlated Single Photon Counting (TCSPC) setup. The samples were excited at 466 nm and their fluorescence was detected at 680 nm. The resulting traces are presented in Fig. 5 and the fitting results are shown in Tab. 2. The H111N mutation significantly shortens the excited state lifetime of CP29 from 2.33 ns in the WT to 1.67 ns. In the C107A/H111N double mutant this effect is even slightly more pronounced, with the excited state lifetime dropping to 1.54 ns. In CP29 H111N, this increased quenching compared to the WT has been attributed to the stronger interaction of the Chl *a*603-*a*609 dimer with the carotenoid in site L2 upon a mutation-induced Chl *a*603 displacement^6^, as the Chl Q_y_-Car S_1_ mixing has been proposed to be a source of Chl excited-state lifetime shortening^69–71^. The observed trend WT-H111N-C107A/H111N in the decrease of the excited state lifetime (see Table 2) agrees with this interpretation, since the proposed increased displacement of Chl *a*603 upon the additional C107A mutation is expected to allow the Chl to get even closer to the carotenoid in site L2 than the H111N single mutant. This mechanism seems to be specific to CP29, since the N47H Lhca4 mutant has a lifetime very similar (10% shorter) to that of Lhca4 WT and the red conformation of the complex was the one identified as being characterized by a longer lifetime^72^.

**Table 2:** Fitting results of the TSCPC data for CP29 WT, H111N, and C107A/H111N

Sample	τ_1 (%)_	τ_2 (%)_	τ_3 (%)_	τ_avg_
CP29 WT	0.21 ns (11%)	1.11 ns (27%)	3.22 ns (62%)	2.33 ns
CP29 H111N	0.11 ns (22%)	0.88 ns (27%)	2.78 ns (51%)	1.67 ns
CP29 C107A/H111N	0.07 ns (24%)	0.80 ns (22%)	2.49 ns (54%)	1.54 ns

In Lhca4 the A43L mutation is sufficient to completely suppress the red-shifted absorption typical of Lhca4, demonstrating that steric perturbations beyond the direct ligation of the bound Chls are key in determining the excitonic properties of the system. The results provide direct experimental evidence that supports our earlier suggestion that the residue at position *i*-4 from the Chl *a*603 ligand influences the Chls *a*603-*a*609 configuration responsible for the red-shifted absorption. In this context, the presence of a small residue at this position appears to favor the geometry required for the formation of the red form. Accordingly, the [A/G]xxx[N] motif emerges as a predictive marker for the presence of red forms in LHCs and may be used to identify far-red–adapted organisms. Targeted mutations of the amino acids at intermediate positions in the motif (labeled as x in the mentioned motif) are not expected to lead to similar effects, since they are not facing the Chls *a*603-*a*609 dimer.

**Fig. 6:**
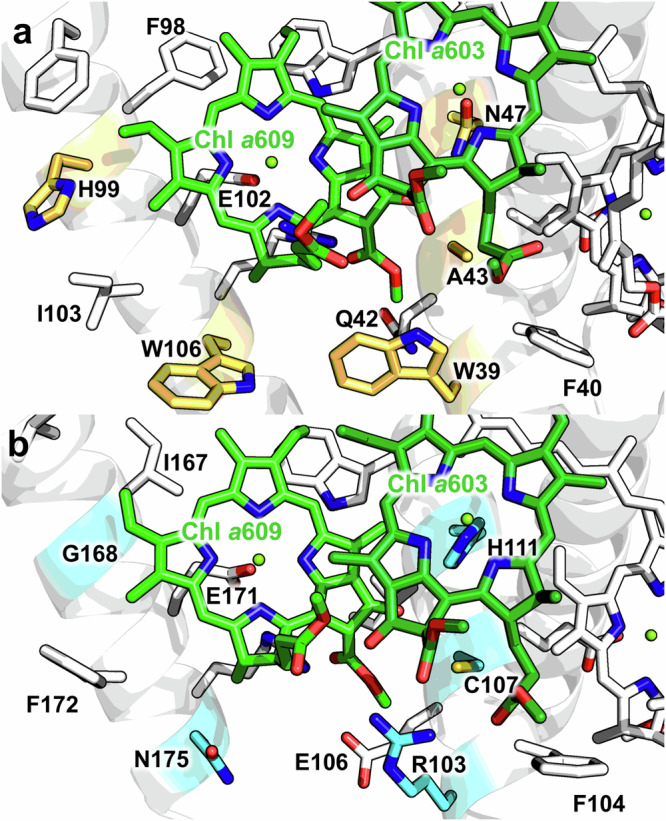
Comparison of the Chl a603-a609 binding site protein environment in Lhca4 and CP29. Schematic view of Chl *a*603–*a*609 pair and the amino acids in van der Waals contact with it in (**a**) *Pisum sativum* Lhca4 (PDB ID: 7DKZ^58^) and (**b**) *Spinacia oleracea* CP29 (PDB ID: 3PL9^59^). Green sticks, Chls *a*; white cartoons, polypeptide chain; yellow and cyan sticks, amino acids mentioned in the text; white sticks, other amino acids and pigments. Phytyl chains and polypeptide backbone have been omitted for clarity.

As shown above, to assess its broader applicability, we investigated whether this motif could be used to modulate the absorption properties of other plant light-harvesting complexes, focusing on CP29. The H111N and C107A/H111N mutants showed a trend towards the appearance of a red-shifted band. The intensity of this band is not comparable to that observed in wild-type Lhca4, suggesting that additional structural elements are required to further red shift the absorption. In particular, differences in the local protein environment surrounding the Chl *a*603–*a*609 pair are likely to limit the extent of the spectral shift. A notable structural difference between the two complexes is the presence in Lhca4 of two tryptophan residues and an histidine in van der Waals contact with the Chl *a*603–*a*609 pair, whereas in CP29 these positions are occupied by an asparagine, an arginine, and a glycine (Fig. 6). Such substitutions, together with other structural differences in the surrounding protein domain, are expected to affect the ability of the binding site to accommodate the altered chlorophyll geometry associated with the red-shifted absorption. In addition, the presence of a positively charged residue (R103) in van der Waals contact with Chl *a*603 in CP29 may disfavor the formation of the *a*603+ *a*609− CT state, which has recently been proposed to contribute substantially to the red form in Lhca4^7,8,41^. Further mutation analysis of these positions coupled with calculations would be highly informative in this regard.

Despite these limitations, the C107A/H111N mutant does exhibit a red-shifted absorption compared to WT CP29. Although modest in magnitude compared to Lhca4 WT, this result provides a proof of principle that the [A/G]xxx[N] motif can be exploited to modulate chlorophyll interactions beyond PSI-associated LHCs.

## Conclusions

Our findings highlight how local steric and electrostatic features of the protein matrix modulate chlorophyll–chlorophyll interactions and thereby tune the low-energy optical properties of chlorophyll clusters. Although the present study does not directly resolve the charge-transfer character of these states, the observed spectral shifts are consistent with changes in the balance between excitonic coupling and charge-transfer contributions proposed for red forms, in line with extensive experimental and theoretical evidence showing that protein electrostatics, hydrogen-bonding networks, and pigment packing can tune the energy and hybridization of charge-transfer states in both antenna and reaction center complexes^7,38–41,73–75^.

Within this established framework, our results show that the residue at the *i*-4 position relative to the Chl *a*603 ligand plays a key role in enabling the red form in Lhca4, as its substitution alone is sufficient to suppress far-red absorption in the Lhca4 A43L mutant. More generally, this position appears to modulate the geometry of the Chl *a*603–*a*609 pair and thereby control access to red-shifted states. Accordingly, the [A/G]xxx[N] motif emerges as a useful design principle to introduce red forms in different light-harvesting complexes. The introduction of far-red states, however, likely requires fine-tuning of the size, polarizability, and electrostatic properties of the amino acids within the binding pocket of the Chl *a*603–*a*609 pair in addition to this motif, as illustrated by the CP29 C107A/H111N case.

Therefore, our insights provide a molecular-level understanding of red-shifted states in pigment–protein complexes and may ultimately inform strategies aimed at extending the photosynthetically active radiation into the far-red, in both artificial and natural systems^76–79^.

## Methods

### Sample preparation

The A43L mutant of Lhca4 was obtained by modifying the pET-28a (+) vector that contained the coding sequence of Lhca4 of *Arabidopsis thaliana*^72^. The H111N and C107A/H111N mutants of CP29 was obtained by modifying the pET-28a (+) vector that contained the coding sequence of CP29 of *A. thaliana*^60^. The apoproteins of the mutants and of the corresponding WT were overexpressed in *Escherichia coli* [BL-21(DE3)] and purified as inclusion bodies. In vitro reconstitution experiments were performed as previously reported^80^. Chls *a*, *b*, and Cars were extracted from spinach leaves^80^. For the reconstitution of the CP29 variants, 800 μg of inclusion body, 160 μg of Cars, and 500 μg of Chls (in a Chl *a*/*b* ratio of 3.0) were used. For the reconstitution of the Lhca4 variants, 800 μg of inclusion body, 160 μg of Cars, and 750 μg of Chls (in a Chl *a*/*b* ratio of 4.0) were used. The Chl *a*/*b* ratio in the pigment mix were chosen to yield recombinant Lhca4 and CP29 complexes with similar pigmentation to their native counterpart. The reconstituted complexes were purified by His-tag Ni-affinity chromatography followed by sucrose density gradient ultracentrifugation with a 0.1–1.0 M sucrose gradient containing 0.06% n-dodecyl-β-D-matloside and 10 mM Hepes at pH 7.5, centrifuging at 41,000 rpm (Beckman Coulter, SW41 rotor) at 4 °C for 17 h^80^.

### Steady-state spectroscopy

Absorption spectra were recorded on a Varian Cary 4000 UV–vis spectrophotometer. For measurements at 77 K, a home-built liquid-nitrogen-cooled device was used. The samples were supplemented with 70% (v/v) of glycerol to obtain an optical quality glass. Emission spectra were recorded on a HORIBA JobinYvon-Spex Fluorolog 3.22 spectrofluorimeter at an optical density of < 0.05 cm^−1^ at the Q_y_ maximum. Circular dichroism (CD) spectra were measured with a Chirascan CD spectrophotometer at 277 and 283 K for Lhca4 and CP29 variants, respectively.

### Pigment composition analysis

The pigments were extracted from the LHCs with 80% acetone. The Chl *a*/*b* and Chl/Car ratios were estimated by fitting the 80% acetone absorption spectrum with the spectra of the individual pigments in the same solvent^81,82^.

### Time-resolved fluorescence

Time-resolved fluorescence measurements were performed using a time-correlated single-photon counting (TCSPC) setup (PicoQuant FluoTime 200) at RT. The optical density of the samples was <0.05 cm^−1^ at the Q_y_ maximum. A laser diode provided the pulsed excitation light at a frequency of 10 MHz and a center wavelength of 466 nm. The instrument response function was determined to be 92 ps (fwhm) measuring the fluorescence decay of a pinacyanol iodide dye dissolved in methanol that has a lifetime of 6 ps. Global analysis of the TCSPC data was performed using the TRFA Data Processor Advanced software^83^, incorporating the experimentally determined IRF.

### Time-resolved absorption

Transient absorption experiments were conducted using a custom-built setup as previously described in ref. ^84^. Briefly, a Coherent MIRA mode-locked Ti:Sa oscillator served as the seed laser for a Coherent Rega 9050 regenerative amplifier, generating ~70 fs pulses centered at 800 nm with a 40 kHz repetition rate. The output beam was divided by a beam splitter, sending 80% of the energy to the pump arm and 20% to the probe arm. The pump beam was converted to 642 nm with a Coherent OPA 9400 optical parametric amplifier, and its spectral bandwidth was narrowed to 10 nm using an interference filter. The probe beam was generated by focusing the regenerative amplifier output into a YAG crystal to produce a white-light supercontinuum. Temporal delay between the pump and probe pulses was adjusted via a retroreflector mounted on a motorized translation stage. Both pump and probe beams could be optically chopped on a shot-to-shot basis using two AA Opto-Electronic acousto-optic modulators, which were synchronized using a Stanford Research Systems DG645 digital delay generator triggered by the amplifier’s repetition rate. This configuration enabled active correction for dark current and scattering. Probe spectra were recorded with a Chromex 250IS spectrograph coupled to an Entwicklungsbüro EB Stresing CCD detector. The relative polarization between pump and probe was fixed to the magic angle (54.7°) using a Berek variable waveplate and a polarizer. Samples were measured in 1 mm cuvettes with an optical density below 0.6 mm^−^^1^ and were continuously stirred during data acquisition. Power-dependent measurements confirmed the absence of exciton–exciton annihilation, and sample stability was verified by comparing emission spectra before and after the transient absorption measurements.

The transient absorption data were analyzed using the pyglotaran Python package^85–87^. In this case, the IRF was not measured directly but modeled as a Gaussian function with a fitted full width at half-maximum (FWHM) of ~100 fs. To focus on the energy transfer dynamics in the transient absorption data, the global analysis was restricted to delays up to 200 ps and to the Q region (λ > 620 nm).

### Visualization

OriginPro 2015 (OriginLab Corporation) was used for plotting the graphs. All structure figures were prepared using PyMol 2.5 (The PyMOL Molecular Graphics System, http://www.pymol.org).

### Reporting summary

Further information on research design is available in the Nature Portfolio Reporting Summary linked to this article.

## Supplementary information

Transparent Peer Review file

Supplemental material

Reporting summary

## Data Availability

All study data are included in the article and supplementary information (SI) and have been made available in Zenodo (10.5281/zenodo.19555493).
