# Supplementary material for: A design principle for tuning far-red absorption of chlorophyll a in light-harvesting complexes
Source: Commun Chem. 2026 May 4;9:238. doi: 10.1038/s42004-026-02052-0 (PMC13347055; doi:10.1038/s42004-026-02052-0)
Supplement: Supplementary file 2 — Supplemental material [file 42004_2026_2052_MOESM2_ESM.pdf]

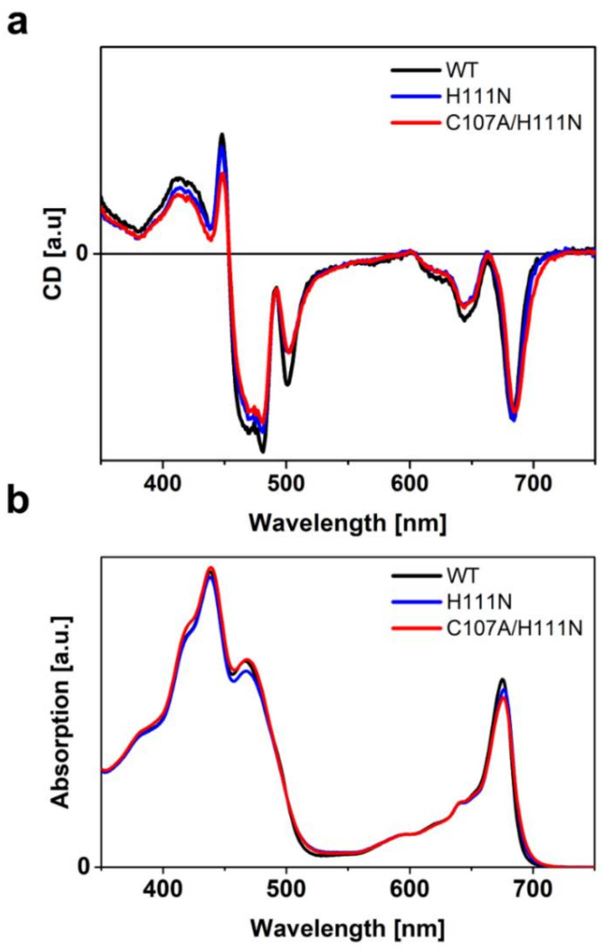

Fig. S1| 283K CD spectra (a) of CP29 WT (black line), H111N (blue line), and H111N/C107A (red line) complexes. Spectra are normalized to the same absorption in the Q region (600-750 nm). Corresponding RT absorption spectra **(b)** are reported for comparison.

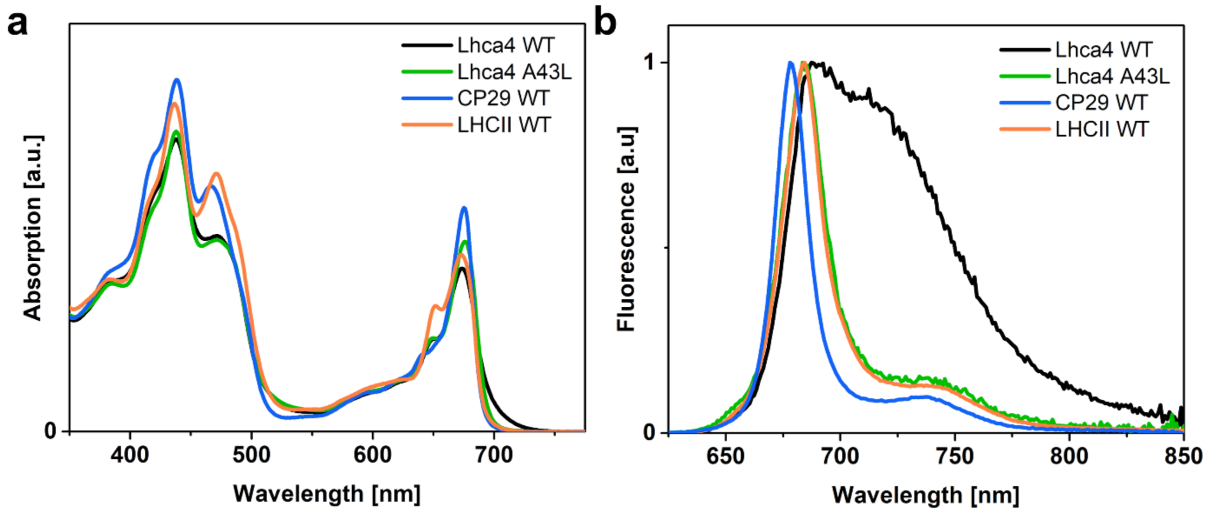

Fig. S2| RT absorption (a) and fluorescence (b) spectra of Lhca4 WT (black) and A43L (green), CP29 WT (blue), and LHCII WT (orange, adapted from Cianfarani et al.[1]). Emission spectra recorded after excitation at 500 nm. Absorption spectra are normalized to the same integrated area in the Q-region (630-750 nm), the fluorescence spectra are normalized to their maxima.

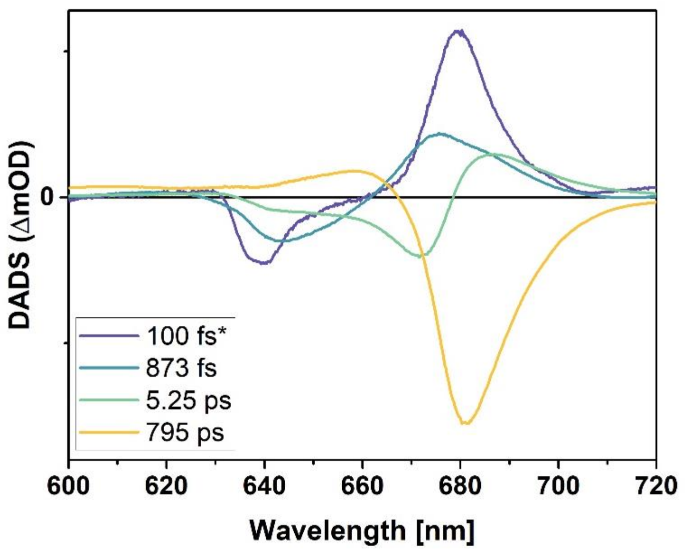

Fig. S3| DADS of CP29 C107A/H111N (extended range of Fig. 4f). The star (*) marks a fixed lifetime.

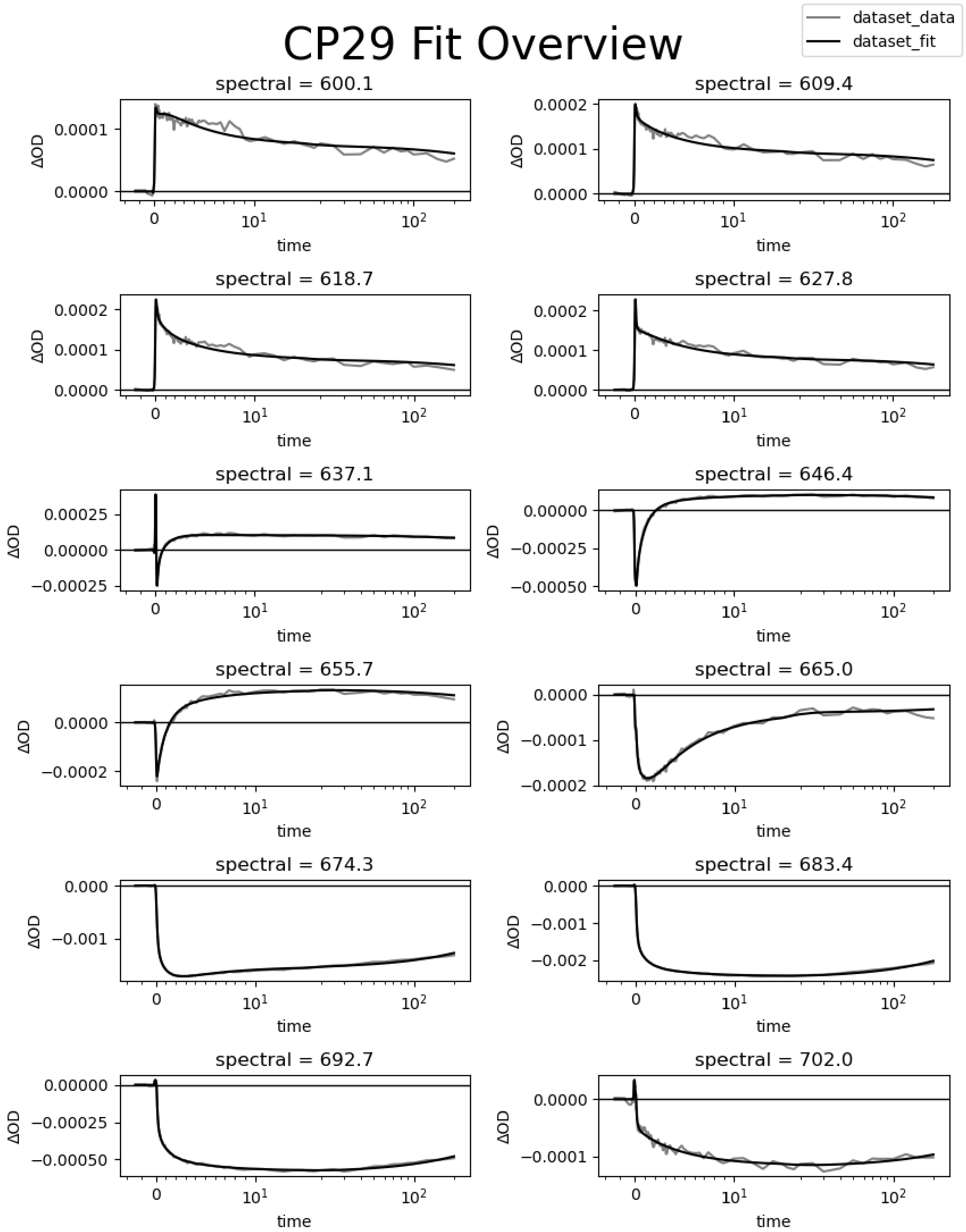

Fig. S4| Fitting quality of the global analysis of the CP29 WT sample. For a selection of wavelengths the time-trace of the raw data of the transient absorption experiment (grey) is shown along the globally fitted trace (black). The time axis is linear until 10 ps and logarithmic thereafter.

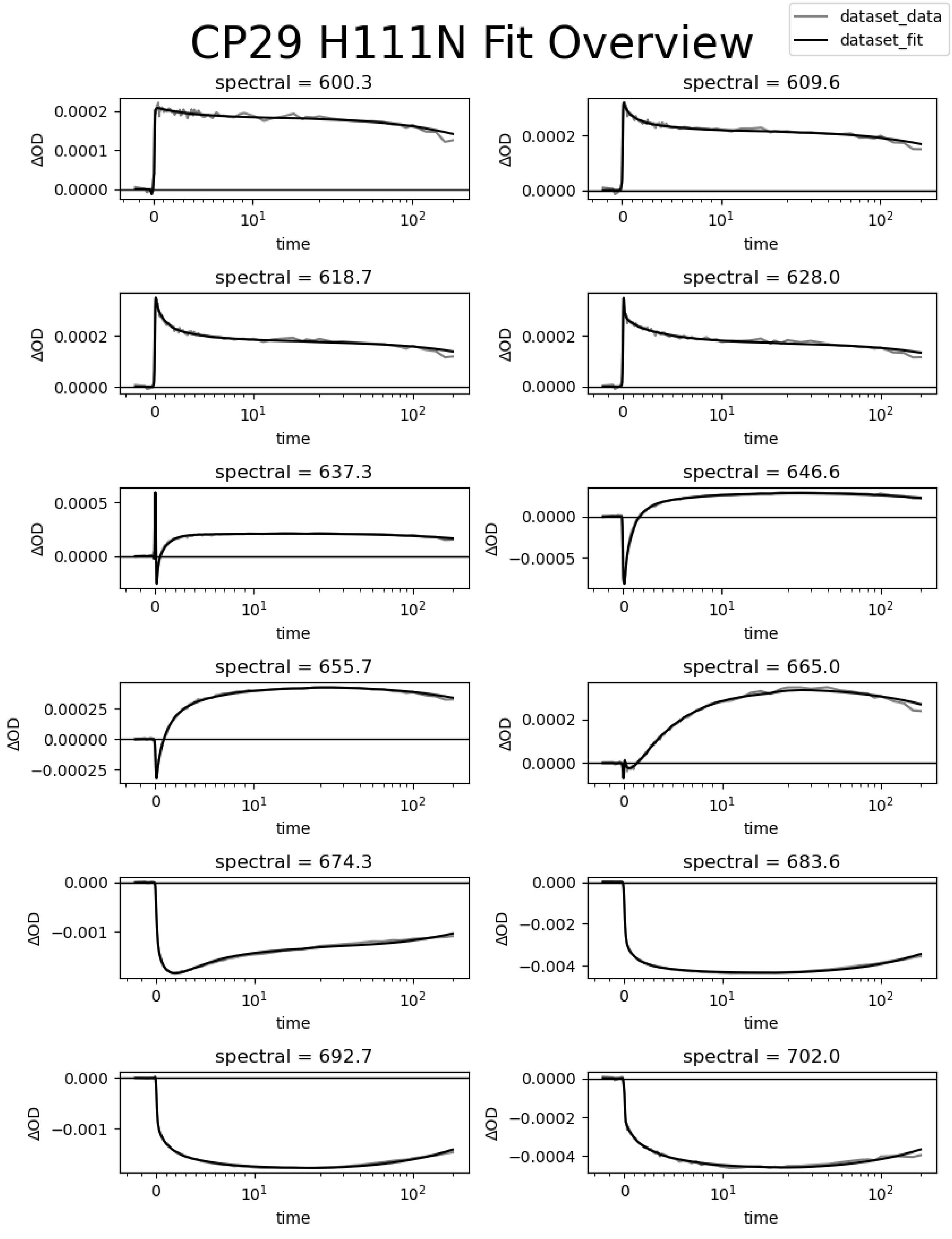

Fig. S5| Fitting quality of the global analysis of the CP29 H111N sample. For a selection of wavelengths the time-trace of the raw data of the transient absorption experiment (grey) is shown along the globally fitted trace (black). The time axis is linear until 10 ps and logarithmic thereafter.

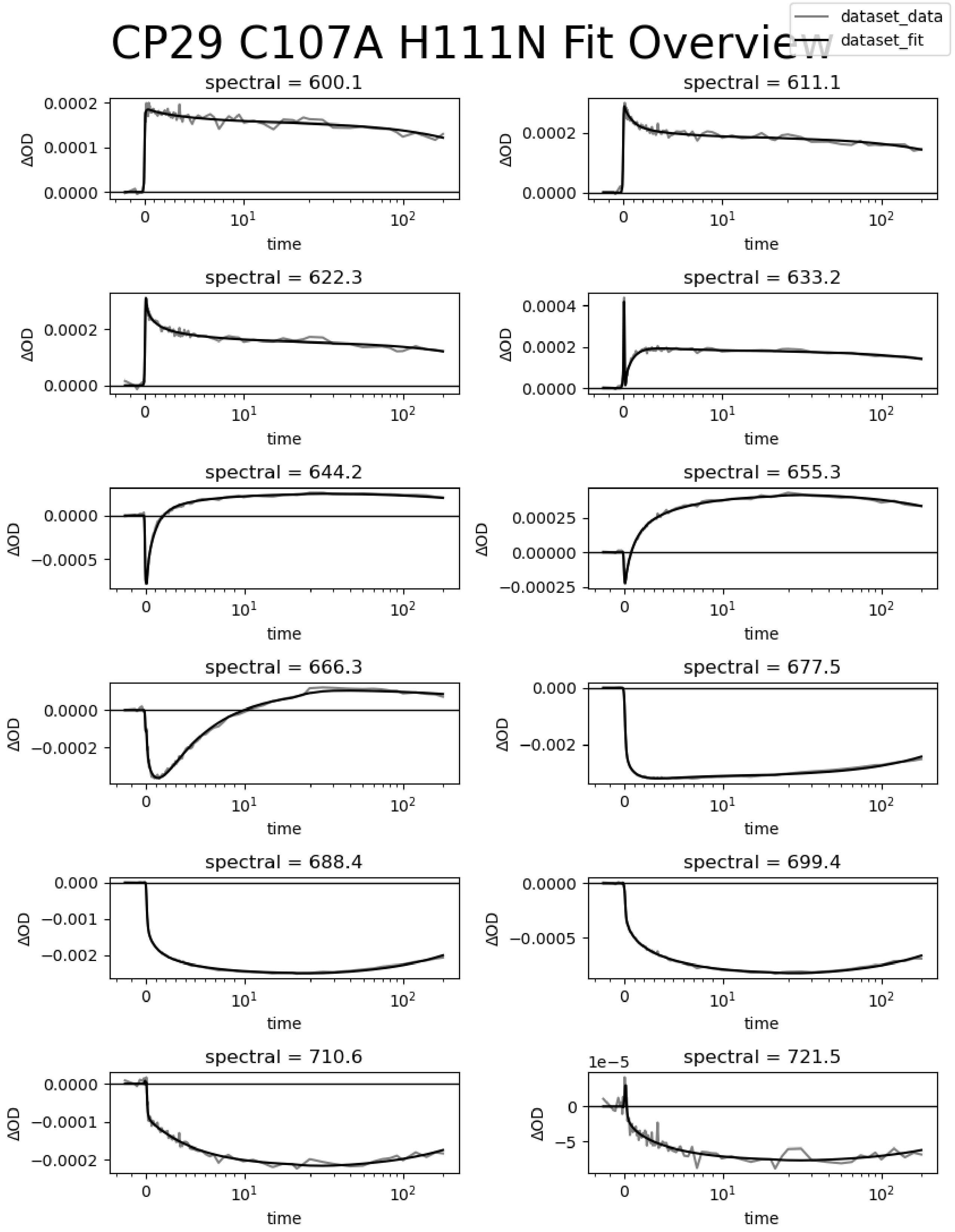

Fig. S6| Fitting quality of the global analysis of the CP29 C107A/H111N sample. For a selection of wavelengths the time-trace of the raw data of the transient absorption experiment (grey) is shown along the globally fitted trace (black). The time axis is linear until 10 ps and logarithmic thereafter.

References

1. Cianfarani, N. *et al.* Far-Red Absorbing LHCII Incorporating Chlorophyll *d* Preserves Photoprotective Carotenoid Triplet–Triplet Energy Transfer Pathways. *J. Phys. Chem. Lett.* **16**, 1720–1728 (2025).
