# Supplementary material for: A design principle for tuning far-red absorption of chlorophyll a in light-harvesting complexes
Source: Commun Chem. 2026 May 4;9:238. doi: 10.1038/s42004-026-02052-0 (PMC13347055; doi:10.1038/s42004-026-02052-0)
Supplement: Supplementary file 1 — Transparent Peer Review file [file 42004_2026_2052_MOESM1_ESM.pdf]

nature portfolio

Peer Review File

A design principle for tuning far-red absorption of chlorophyll *a* in light-harvesting complexes

Corresponding Author: Dr Alessandro Agostini

This file contains all reviewer reports in order by version, followed by all author rebuttals in order by version.

Version 0:

Reviewer comments:

Reviewer #1

(Remarks to the Author)
In their work, Agostini et al. test the recent hypothesis that the properties of the residue located at the i-4 position from the ligand play a crucial role in formation of the far-red states in various photosynthetic light-harvesting complexes. In particular, they perform a targeted mutagenesis on the Lhca4 and CP29 complexes—the former normally exhibiting a wide absorption sideband extending beyond 700 nm together with a strong emission in this spectral region, and the later lacking these properties.
The work presents interesting results of targeted gene manipulation combined with the steady-state and time-resolved spectroscopic measurements of the reconstituted pigment–protein complexes. However, I have some concerns regarding the novelty of some of the presented results and some aspects of their interpretations that should be clarified before the paper can be published:

1. Somewhat similar measurements of time-resolved fluorescence and transient absorption of the H111N mutant of CP29 were performed recently by Sardar et al. (Ref. 8). While their obtained spectra qualitatively resemble those presented in the current work, there are some differences in the obtained lifetimes that should be clearly addressed.

2. The authors suggest that the [A/G]xxxN pattern can be a predictive marker for the red forms in LHCs. However, there is a significant difference between the probable nature of the “red forms” in Lhca4 WT and CP29 mutant: in CP29, it does not seem that any new state is formed, the spectral redshift is rather small, and the main effect seems to be the increased disorder (leading to the broader spectrum) and more intense vibrational sideband in FL spectrum. This effect can hardly be called the "far-red absorption", as the title suggests. On the other hand, the far-red band in Lhca4 is believed to arise from the inter-chlorophyll charge transfer involving Chl a603. As such, it, of course, depends on the excitonic inter-chlorophyll interactions, but is far more sensitive to the local electrostatics. Could it be that A43L mutation promotes the protonation of the otherwise negative nearby Glu44, so that the strong electric field in this Chl binding pocket disappears, making the charge transfer much less probable?

3. Could other targeted mutations of the aminoacids between A and N (labeled as x in the mentioned motif) lead to similar effects? Such possibilities should also be considered/discussed. While I agree that the presented results nicely demonstrate how subtle tuning in local environment can affect the inter-molecular interactions, I am not convinced that it is this specific AxxxN motif that ensures the far red fluorescence. Thus either more elaborate evidence must be presented, or the proposed claims should be softened.

4. Some minor remarks:
a. Some clarification on whether WT samples were just purified from leaves or reconstituted in a similar way as mutants would be helpful.
b. Lines 110–111: what does it mean “the concentration < 0.05 cm–1”?
c. Lines 213 and 215: it should be Fig. 4 instead of Fig. 5.
d. Legend of Fig. 4f: what does the star (*) after “100 fs” mean?
e. Supplementary: Fig. S1b is mentioned, but not referred to in the figure caption.

Reviewer #2

(Remarks to the Author)
The manuscript originates from a well-reputed and widely recognized research group specialized in investigations of the mechanisms governing the functioning of photosynthetic antennae. The manuscript corresponds very well with a whole series of other works of this group. The aim of the manuscript, i.e., to reveal the principles of fine tuning of the electronic structure of chlorophyll a clusters for absorption in the far-red region of the spectrum, is sound and clearly stated. The entire study is well designed and performed equally well with the use of adequate methodology, both in terms of sample preparation and analysis of the impact of mutations introduced in the CP29 polypeptide, based on advanced spectroscopic methods. Also, the manuscript is well organized, clearly written, and reads very well. The conclusions drawn by the authors are well evidenced by the results. A particularly interesting is the identification of a specific motif ([A/G]xxx[N]) to be a predictive marker of polypeptide sequences responsible for environmental tuning of chlorophyll a dimers/clusters for light absorption in the far-red. Surely, further investigations are required to show how universal it is, but the present result is already meaningful and will be of broad interest. To this end, I have a very minor remark. Namely, it is concluded (l. 282) that the charge transfer character of Chl clusters is among the factors that contribute to such tuning. However, the present study did not really address this aspect and does not provide much support for this conclusion. I suggest briefly clarifying this point or rephrase it accordingly.
In conclusion, my recommendation is to publish the manuscript in the present form, with a very minor revision.

Reviewer #3

(Remarks to the Author)
This is a strong, hypothesis-driven study that makes a clear mechanistic contribution to our understanding of far-red absorption in eukaryotic light-harvesting complexes. By combining targeted mutagenesis with spectroscopy in two structurally related but functionally distinct antenna proteins (Lhca4 and CP29), the authors convincingly show that steric constraints near the Chl a603 ligand, specifically at the i-4 position, are a key determinant of red-shifted absorption. The work advances the field by moving beyond correlation and toward a design principle. The choice of mutations is thoughtful and mechanistically meaningful. Sufficient details are provided to enable reproduction of this work by other researchers.

A central conclusion of the manuscript is that steric crowding destabilizes a specific pigment configuration, thereby modulating excitonic and CT interactions. While this interpretation is plausible and consistent with the observed spectroscopic trends, it is inferred rather than directly demonstrated. In particular, no structural data are provided to confirm how pigment orientations, distances, or relative geometries change upon mutation. As a result, it remains unclear whether the reported effects arise from the proposed steric rearrangements of the pigments, from altered electrostatics, or from more subtle, indirect perturbations of the protein environment. The authors could substantially strengthen this claim by either providing structural evidence (e.g., high-resolution structures or validated structural models) or by more explicitly acknowledging the inferential nature of the steric argument and discussing alternative explanations. Even coarse-grained modeling or restrained MD could have strengthened the causal link between steric changes and pigment geometry.

The manuscript emphasizes the role of local steric and electrostatic features of the protein matrix in shaping excitonic–charge-transfer mixing in chlorophyll clusters. While this interpretation is physically well founded, the central importance of local packing and electrostatic environment for exciton–CT hybridization has long been established in the organic semiconductor and OPV literature. As currently phrased, the claim risks appearing descriptive rather than conceptually novel. The authors could strengthen the impact of this section by clarifying what is new relative to existing frameworks, e.g., whether the protein matrix induces a qualitatively different regime of exciton–CT coupling, enables unusually strong or weak mixing, or allows disentangling steric and electrostatic contributions in a way not previously demonstrated. Explicitly positioning the results with respect to prior work on environmental control of CT states would help delineate the specific advance made here.

Several statements in the manuscript appear overstated and would benefit from more precise wording. For example, in lines 161–162 the claim that absorption above 700 nm is “completely suppressed” is not strictly supported by the data, as Fig. 2(a) shows a residual absorption tail beyond 700 nm. This suggests that the far-red emission isn’t completely lost, either on average or for all complexes. The former can be justified by the fact that static disorder may occasionally favor pigment configurations that still allow some degree of exciton–CT mixing in the 603-609 dimer, so that some fraction of complexes would always exhibit some far-red absorption, consistent with earlier single-molecule studies on Lhca4 (https://doi.org/10.1073/pnas.110541110).

A similar concern applies to the statement in line 167 that far-red emission is “completely lost.” It would be helpful to justify this claim more rigorously, for example by comparison with reference spectra from CP29 or LHCII, or to clarify whether a small residual far-red emission may still be present. Related language on lines 22 and 251 may also need to be moderated to avoid absolute claims that are not fully supported by the data.

In lines 211–213, the manuscript focuses on a slight increase in the red tail of the double mutant; however, this is not the only spectral difference. In (h), the single mutant also exhibits an enhanced red tail, and in both panels (g) and (h) there are clear peak shifts around ~640 nm and ~680 nm. These additional spectral changes should be explicitly discussed, as they may provide further insight into the underlying pigment–pigment interactions.

Prior single-molecule spectroscopy studies reporting far-red emission from CP29 can be cited as circumstantial support for the presence of environmentally sensitive, low-energy states in this complex, which may help contextualize the present results.

In line 260, the phrase “we next tested” suggests the introduction of new results, whereas the text refers to data already presented. Rephrasing would improve clarity and avoid potential confusion.

The manuscript contains a relatively high proportion of self-citations (29 out of 79 references, ~37%). While some are clearly relevant, the authors may wish to consider whether all are necessary or whether additional independent literature could be cited where appropriate.

Fig. S1’s caption lacks the label (b).

Ref. 44 lacks details.

Version 1:

Reviewer comments:

Reviewer #1

(Remarks to the Author)
In their updated manuscript, authors has extended the description of the methodology and the discussion of the obtained results along with the reviewer's suggestions. After these clarifications, the paper is now ready for publication.
Before the publication, however, the newly added structural Fig. 6 should be checked: it contains only two panels (a and b), while its caption additionally refers to panel c with LHCII structure.

Reviewer #3

(Remarks to the Author)
The revised manuscript is now more carefully phrased and better contextualized, and the cross-complex analysis of the [A/G]xxxN motif strengthens the structure–function narrative. However, for a broad-scope journal such as Communications Chemistry, the conceptual advance would benefit from being articulated more explicitly: in particular, it remains somewhat unclear whether the work establishes a new mechanistic regime of exciton–CT mixing or primarily provides system-specific support for established environmental tuning principles. Clarifying more directly what transferable design rule emerges beyond prior CT–environment frameworks would further sharpen the impact of the study.

Version 2:

Reviewer comments:

Reviewer #3

(Remarks to the Author)
The authors have satisfactorily addressed my concern, and I support publication of the manuscript.

Open Access This Peer Review File is licensed under a Creative Commons Attribution 4.0 International License, which permits use, sharing, adaptation, distribution and reproduction in any medium or format, as long as you give appropriate credit to the original author(s) and the source, provide a link to the Creative Commons license, and indicate if changes were made.
In cases where reviewers are anonymous, credit should be given to 'Anonymous Referee' and the source.
The images or other third party material in this Peer Review File are included in the article's Creative Commons license, unless indicated otherwise in a credit line to the material. If material is not included in the article's Creative Commons license and your intended use is not permitted by statutory regulation or exceeds the permitted use, you will need to obtain permission directly from the copyright holder.
To view a copy of this license, visit https://creativecommons.org/licenses/by/4.0/

Reviewer #1 (Remarks to the Author):

In their work, Agostini et al. test the recent hypothesis that the properties of the residue located at the i-4 position from the ligand play a crucial role in formation of the far-red states in various photosynthetic light-harvesting complexes. In particular, they perform a targeted mutagenesis on the Lhca4 and CP29 complexes—the former normally exhibiting a wide absorption sideband extending beyond 700 nm together with a strong emission in this spectral region, and the later lacking these properties.

The work presents interesting results of targeted gene manipulation combined with the steady-state and time-resolved spectroscopic measurements of the reconstituted pigment–protein complexes. However, I have some concerns regarding the novelty of some of the presented results and some aspects of their interpretations that should be clarified before the paper can be published:

1. Somewhat similar measurements of time-resolved fluorescence and transient absorption of the H111N mutant of CP29 were performed recently by Sardar et al. (Ref. 8). While their obtained spectra qualitatively resemble those presented in the current work, there are some differences in the obtained lifetimes that should be clearly addressed.

Answer: The LHCs exist in different conformations characterized by distinct lifetimes. In the presence of red forms, this equilibrium becomes very sensitive to the environment. The difference can then be explained by the fact that our samples are in detergent while in Sardar et al. they used nanodisk. However, the difference is only 0.3 ns, which is not particularly significant.

2. The authors suggest that the [A/G]xxxN pattern can be a predictive marker for the red forms in LHCs. However, there is a significant difference between the probable nature of the "red forms" in Lhca4 WT and CP29 mutant: in CP29, it does not seem that any new state is formed, the spectral redshift is rather small, and the main effect seems to be the increased disorder (leading to the broader spectrum) and more intense vibrational sideband in FL spectrum. This effect can hardly be called the "far-red absorption", as the title suggests. On the other hand, the far-red band in Lhca4 is believed to arise from the inter-chlorophyll charge transfer involving Chl a603. As such, it, of course, depends on the excitonic inter-chlorophyll interactions, but is far more sensitive to the local electrostatics. Could it be that A43L mutation promotes the protonation of the otherwise negative nearby Glu44, so that the strong electric field in this Chl binding pocket disappears, making the charge transfer much less probable?

Answer: Certainly the environment can change the pKa of residues, however, our experiments are done at pH 7.5, which is a very high value for glutamate. Moreover, the sidechain of Glu44 is facing away from the A/L43 residue and the *a*603-*a*609 dimer, and kept in position by being the Mg-ligand of Chl *a*602 (see figure 6). Since the amino acid substitution is between two aliphatic amino acids, a role of this mutation in affecting the protonation of Glu44 seems unlikely.

We agree with the reviewer that inter-chlorophyll charge transfer states are expected to be sensitive to the local electrostatics, and in fact the rather small red shift in CP29 H111N/C107A is discussed analyzing the differences in the local protein environment surrounding the Chl a603–a609 pair in CP29 and Lhca4 and how they could affect the stabilization of the CT state. Prompted by the comment of the reviewer, we decided to add emphasis to this section, adding a dedicated figure (Figure 6), that is expected to ease the reader in evaluate the protein environment surrounding of the Chl *a*603–*a*609 pair.

3. Could other targeted mutations of the aminoacids between A and N (labeled as x in the mentioned motif) lead to similar effects? Such possibilities should also be considered/discussed. While I agree that the presented results nicely demonstrate how subtle tuning in local environment can affect the inter-molecular interactions, I am not convinced that it is this specific AxxxN motif that ensures the far red fluorescence. Thus either more elaborate evidence must be presented, or the proposed claims should be softened.

Answer: We specifically targeted the amino acid in position i-4 since in α-helix, residues at positions *i* and *i*–4 occupy approximately the same helical face (since an α-helix contains ~3.6 residues per turn). Thus, the mentioned residues in between are not facing the dimer directly, and their influence on the dimer properties is expected to be of lesser intensity, if any. We have added a more detailed discussion on this point at pages 12-13:

"Accordingly, the [A/G]xxx[N] motif emerges as a predictive marker for the presence of red forms in LHCs and may be used to identify far-red–adapted organisms. Targeted mutations of the amino acids at intermediate positions in the motif (labeled as x in the mentioned motif) are not expected to lead to similar effects, since they are not facing the Chls a603-a609 dimer."

4. Some minor remarks:
a. Some clarification on whether WT samples were just purified from leaves or reconstituted in a similar way as mutants would be helpful. Added at page 6: *"In the present study, both*

WT and mutant proteins were expressed, purified, and reconstituted in vitro under identical conditions to ensure direct comparability."

b. Lines 110–111: what does it mean "the concentration < 0.05 cm–1"? OD at max Q_y, it has been rephrased to improve the clarity.

c. Lines 213 and 215: it should be Fig. 4 instead of Fig. 5. Thank you.

d. Legend of Fig. 4f: what does the star (*) after "100 fs" mean? Fixed lifetime. Added to the figure caption.

e. Supplementary: Fig. S1b is mentioned, but not referred to in the figure caption. Thank you.

Reviewer #2 (Remarks to the Author):

The manuscript originates from a well-reputed and widely recognized research group specialized in investigations of the mechanisms governing the functioning of photosynthetic antennae. The manuscript corresponds very well with a whole series of other works of this group. The aim of the manuscript, i.e., to reveal the principles of fine tuning of the electronic structure of chlorophyll a clusters for absorption in the far-red region of the spectrum, is sound and clearly stated. The entire study is well designed and performed equally well with the use of adequate methodology, both in terms of sample preparation and analysis of the impact of mutations introduced in the CP29 polypeptide, based on advanced spectroscopic methods. Also, the manuscript is well organized, clearly written, and reads very well. The conclusions drawn by the authors are well evidenced by the results. A particularly interesting is the identification of a specific motif ([A/G]xxx[N]) to be a predictive marker of polypeptide sequences responsible for environmental tuning of chlorophyll a dimers/clusters for light absorption in the far-red. Surely, further investigations are required to show how universal it is, but the present result is already meaningful and will be of broad interest. To this end, I have a very minor remark. Namely, it is concluded (l. 282) that the charge transfer character of Chl clusters is among the factors that contribute to such tuning. However, the present study did not really address this aspect and does not provide much support for this conclusion. I suggest briefly clarifying this point or rephrase it accordingly. In conclusion, my recommendation is to publish the manuscript in the present form, with a very minor revision.

Answer: thank you for the nice comments. We have now clarified this point in the conclusions, that now reads: *"Our findings highlight how local steric and electrostatic features of the protein matrix modulate chlorophyll–chlorophyll interactions and thereby tune the low-energy optical properties of chlorophyll clusters. Although the present study does not directly resolve the charge-transfer character of these states, the observed spectral shifts are consistent with changes*

in the balance between excitonic coupling and charge-transfer contributions proposed for red forms, in line with extensive experimental and theoretical evidence showing that protein electrostatics, hydrogen-bonding networks, and pigment packing can tune the energy and hybridization of charge-transfer states in both antenna and reaction center complexes.[7,38–41,81–83]*"*

Reviewer #3 (Remarks to the Author):

This is a strong, hypothesis-driven study that makes a clear mechanistic contribution to our understanding of far-red absorption in eukaryotic light-harvesting complexes. By combining targeted mutagenesis with spectroscopy in two structurally related but functionally distinct antenna proteins (Lhca4 and CP29), the authors convincingly show that steric constraints near the Chl a603 ligand, specifically at the i-4 position, are a key determinant of red-shifted absorption. The work advances the field by moving beyond correlation and toward a design principle. The choice of mutations is thoughtful and mechanistically meaningful. Sufficient details are provided to enable reproduction of this work by other researchers.

A central conclusion of the manuscript is that steric crowding destabilizes a specific pigment configuration, thereby modulating excitonic and CT interactions. While this interpretation is plausible and consistent with the observed spectroscopic trends, it is inferred rather than directly demonstrated. In particular, no structural data are provided to confirm how pigment orientations, distances, or relative geometries change upon mutation. As a result, it remains unclear whether the reported effects arise from the proposed steric rearrangements of the pigments, from altered electrostatics, or from more subtle, indirect perturbations of the protein environment. The authors could substantially strengthen this claim by either providing structural evidence (e.g., high-resolution structures or validated structural models) or by more explicitly acknowledging the inferential nature of the steric argument and discussing alternative explanations. Even coarse-grained modeling or restrained MD could have strengthened the causal link between steric changes and pigment geometry.

Answer: We thank the Reviewer for this insightful suggestion. We agree that direct structural data or dedicated computational modeling would further strengthen the causal link between steric perturbations and pigment geometry. However, such approaches would require substantial additional work and are beyond the scope of the present hypothesis-driven mutagenesis and spectroscopy study. We have included a new structural figure (Fig. 6) and an expanded analysis of the local environment surrounding the Chl a603–a609 pair, in order to strengthen the mechanistic framework of the manuscript while providing a solid basis for future mutagenesis and computational investigations.

The manuscript emphasizes the role of local steric and electrostatic features of the protein matrix in shaping excitonic–charge-transfer mixing in chlorophyll clusters. While this interpretation is physically well founded, the central importance of local packing and electrostatic environment for exciton–CT hybridization has long been established in the organic semiconductor and OPV literature. As currently phrased, the claim risks appearing descriptive rather than conceptually novel. The authors could strengthen the impact of this section by clarifying what is new relative to existing frameworks, e.g., whether the protein matrix induces a qualitatively different regime of exciton–CT coupling, enables unusually strong or weak mixing, or allows disentangling steric and electrostatic contributions in a way not previously demonstrated. Explicitly positioning the results with respect to prior work on environmental control of CT states would help delineate the specific advance made here.

Answer: we clarified by an extensive rephrasing of the conclusions, that now reads: *"Our findings highlight how local steric and electrostatic features of the protein matrix modulate chlorophyll–chlorophyll interactions and thereby tune the low-energy optical properties of chlorophyll clusters. Although the present study does not directly resolve the charge-transfer character of these states, the observed spectral shifts are consistent with changes in the balance between excitonic coupling and charge-transfer contributions proposed for red forms, in line with extensive experimental and theoretical evidence showing that protein electrostatics, hydrogen-bonding networks, and pigment packing can tune the energy and hybridization of charge-transfer states in both antenna and reaction center complexes.[7,38–41,81–83]*

Therefore, our insights provide a molecular-level understanding of red-shifted states in pigment–protein complexes and may ultimately inform strategies aimed at extending the photosynthetically active radiation into the far-red, in both artificial and natural systems.[84–87]"

Several statements in the manuscript appear overstated and would benefit from more precise wording. For example, in lines 161–162 the claim that absorption above 700 nm is "completely suppressed" is not strictly supported by the data, as Fig. 2(a) shows a residual absorption tail beyond 700 nm. This suggests that the far-red emission isn't completely lost, either on average or for all complexes. The former can be justified by the fact that static disorder may occasionally favor pigment configurations that still allow some degree of exciton–CT mixing in the 603-609 dimer, so that some fraction of complexes would always exhibit some far-red absorption, consistent with earlier single-molecule studies on Lhca4 (https://doi.org/10.1073/pnas.110541110).

Answer: What we mean is that the absorption above 700 nm is at the same level observed in other LHCs that do not contain red forms, such as LHCII. We have prepared a new figure (Fig.

S2) to better convey this. However, we agree with the reviewer, and indeed occasionally other LHCs also show far-red emission at the single-molecule level, which probably stems from a small population of complexes with some degree of exciton–CT mixing. This is now explained more clearly in the manuscript at pages 6-7: *"The small residual absorption tail beyond 700 nm is at the same level observed in other LHCs that do not contain red forms, such as LHCII (Fig. S2a). Static disorder may occasionally favor pigment configurations that still allow some degree of exciton–CT mixing in the a603-a609 dimer, so that some fraction of complexes would always exhibit some far-red absorption, consistent with earlier single-molecule studies on Lhca4[72] and related LHCs[73–77]."*

A similar concern applies to the statement in line 167 that far-red emission is "completely lost." It would be helpful to justify this claim more rigorously, for example by comparison with reference spectra from CP29 or LHCII, or to clarify whether a small residual far-red emission may still be present. Related language on lines 22 and 251 may also need to be moderated to avoid absolute claims that are not fully supported by the data.

Answer: We added a figure with the comparison suggested by the reviewer in the supporting info (Figure S2), from which it clearly appears that the emission of the Lhca4 A43L mutant is superimposable to the one of LHCII WT. Therefore, the language at line 251 has been retained, since it is based on the collected data. Line 22 has been moderated as suggested, since it refers instead to the absorption (that has been discussed in detail, see previous feedback).

In lines 211–213, the manuscript focuses on a slight increase in the red tail of the double mutant; however, this is not the only spectral difference. In (h), the single mutant also exhibits an enhanced red tail, and in both panels (g) and (h) there are clear peak shifts around ~640 nm and ~680 nm. These additional spectral changes should be explicitly discussed, as they may provide further insight into the underlying pigment–pigment interactions.

Answer: We added the suggested description, and the section now reads: " The first and second components, dominated by the energy transfer from Chls b (640-650 nm) to Chls a (670-685 nm), have similar lifetime and spectra in all three CP29 variants, with peak red-shifts and a slight increase in the red tail that follow the trend WT<H111N<C107A/H111N (Fig. 4g-h)."

Prior single-molecule spectroscopy studies reporting far-red emission from CP29 can be cited as circumstantial support for the presence of environmentally sensitive, low-energy states in this complex, which may help contextualize the present results.

Answer: As suggested, we contextualized our results with relevant literature dealing with single-molecule spectroscopy of Lhca4 [ref. 72 in the revised manuscript] and related LHCs [refs. 73-77 in the revised manuscript].

In line 260, the phrase "we next tested" suggests the introduction of new results, whereas the text refers to data already presented. Rephrasing would improve clarity and avoid potential confusion.

Answer: We rephrased it as follow: *"As shown above, to assess its broader applicability, we investigated"*

The manuscript contains a relatively high proportion of self-citations (29 out of 79 references, ~37%). While some are clearly relevant, the authors may wish to consider whether all are necessary or whether additional independent literature could be cited where appropriate.

Answer: We appreciate the reviewer's comment regarding the proportion of self-citations. We agree that self-citation is inappropriate when references are not clearly relevant. However, the cited studies from our group constitute key methodological and conceptual foundations of this work, and our laboratory has played a central role in developing this research field. Omitting these references would not accurately reflect the development of the field or the basis of the present study.

We have nonetheless re-examined the reference list and ensured that all self-citations are necessary and directly relevant, and we have added additional independent references where appropriate.

Fig. S1's caption lacks the label (b).

Answer: thanks

Ref. 44 lacks details.

Answer: fixed

In the following reply letter to the reviewers, you will find our point-by-point responses to the reviewers' comments. Therein, reviewers' comments are printed in black, and our response in blue. All modifications are also visible in yellow in a marked-up version of the manuscript.

Reviewers' comments:

Reviewer #1 (Remarks to the Author):

In their updated manuscript, authors has extended the description of the methodology and the discussion of the obtained results along with the reviewer's suggestions. After these clarifications, the paper is now ready for publication.

Before the publication, however, the newly added structural Fig. 6 should be checked: it contains only two panels (a and b), while its caption additionally refers to panel c with LHCII structure.

Answer: Fixed, thank you.

Reviewer #3 (Remarks to the Author):

The revised manuscript is now more carefully phrased and better contextualized, and the cross-complex analysis of the [A/G]xxxN motif strengthens the structure–function narrative. However, for a broad-scope journal such as Communications Chemistry, the conceptual advance would benefit from being articulated more explicitly: in particular, it remains somewhat unclear whether the work establishes a new mechanistic regime of exciton–CT mixing or primarily provides system-specific support for established environmental tuning principles. Clarifying more directly what transferable design rule emerges beyond prior CT–environment frameworks would further sharpen the impact of the study.

Answer: We thank the Reviewer for this important comment. Our intention is not to claim a new mechanistic regime of exciton–CT mixing, which is indeed well established, but rather to experimental validate a sequence-level structural determinant that controls access to such regime in chlorophyll-binding proteins. Specifically, our results define the residue at the *i*-4 position relative to the Chl *a*603 ligand as a key steric modulator of the Chl *a*603–*a*609 geometry, and thus of the emergence of red-shifted states.

In the revised manuscript, we have clarified this point by explicitly framing our contribution as a transferable design rule linking local protein sequence to pigment–pigment configuration and spectral tuning, rather than as a new physical mechanism. We also emphasize that the proposed [A/G]xxx[N] motif provides a predictive and engineerable handle to identify or modulate red forms across different LHC families.

The second half of the conclusions now read: *"Within this established framework, our results show that the residue at the i-4 position relative to the Chl a603 ligand plays a key role in enabling the red form in Lhca4, as its substitution alone is sufficient to suppress far-red absorption in the Lhca4 A43L mutant. More generally, this position appears to modulate the geometry of the Chl a603–a609 pair and thereby control access to red-shifted states.*

Accordingly, the [A/G]xxx[N] motif emerges as a useful design principle to introduce red forms in different light-harvesting complexes. The introduction of far-red states, however, likely requires fine-tuning of the size, polarizability, and electrostatic properties of the amino acids within the binding pocket of the Chl a603–a609 pair in addition to the motif, as illustrated by the CP29 C107A/H111N case. Therefore, our insights provide a molecular-level understanding of red-shifted states in pigment–protein complexes and may ultimately inform strategies aimed at extending the photosynthetically active radiation into the far-red, in both artificial and natural systems.[84–87]"

We believe this clarification better delineates the conceptual advance with respect to existing CT–environment frameworks.
